# RE-LOCK and TRACK: A Reproducible Minimally Invasive Repair for Bileaflet Mitral Disease

**DOI:** 10.1093/ejcts/ezag211

**Published:** 2026-08-20

**Authors:** Giuseppe Nasso, Giuseppe Speziale

**Affiliations:** Department of Cardiac Surgery, Santa Maria Hospital, GVM Care & Research, 70124 Bari, Italy; Department of Medicine and Surgery, LUM University, Casamassima, 70010 Bari, Italy; Department of Cardiac Surgery, Santa Maria Hospital, GVM Care & Research, 70124 Bari, Italy; Department of Health and Life Sciences, European University of Rome, 00163 Rome, Italy

**Keywords:** mitral valve repair; Barlow disease; bileaflet prolapse; neochordae; RE-LOCK; TRACK

## Abstract

Complex bileaflet and Barlow-type degenerative disease remains among the most demanding substrates in reconstructive mitral surgery, and a right mini-thoracotomy amplifies every source of intraoperative variability. Two determinants dominate durability: excessive posterior leaflet height, which displaces the coaptation line anteriorly and drives residual regurgitation and systolic anterior motion; and, in anterior prolapse, neochordal length, where a millimetric error produces residual prolapse, restriction or recurrence. We describe RE-LOCK and TRACK, a two-component, posterior-first protocol designed to control both reproducibly. RE-LOCK relocates the redundant posterior leaflet head towards the annular plane and anchors it to a stable posterior reference, converting a mobile prolapsing edge into a controlled hinge and establishing a low, posteriorised coaptation shelf; posterior neochordae are not used. TRACK then calibrates anterior support: expanded-polytetrafluoroethylene neochordae anchored to the papillary head are advanced against a temporary annular guide kept coplanar with the annulus, so that length is set against an anatomical reference rather than by visual estimation or ventricular loading. Complete ring annuloplasty stabilises the result, and intraoperative transoesophageal echocardiography governs any revision. By fixing the sequence, platform first and calibration second, the protocol converts individually demanding manoeuvres into a repeatable, teachable operation particularly well suited to the minimally invasive field.

Complex bileaflet and Barlow-type degenerative disease is among the most demanding substrates in reconstructive mitral surgery, where durable repair—preserving the subvalvular apparatus and left ventricular geometry—remains the goal.^1^ Through a right mini-thoracotomy, limited exposure amplifies every source of intraoperative variability. Two pitfalls in bileaflet repair may compromise durability: leaving excessive height of the posterior leaflet, which displaces the coaptation line anteriorly and drives residual regurgitation and systolic anterior motion; and inappropriately sized anterior chordae, in which a small error in length may produce residual prolapse, restriction or recurrence. RE-LOCK and TRACK is a 2-component, posterior-first protocol designed to control both reproducibly, in the minimally invasive field (Figure 1).

**Figure 1.**
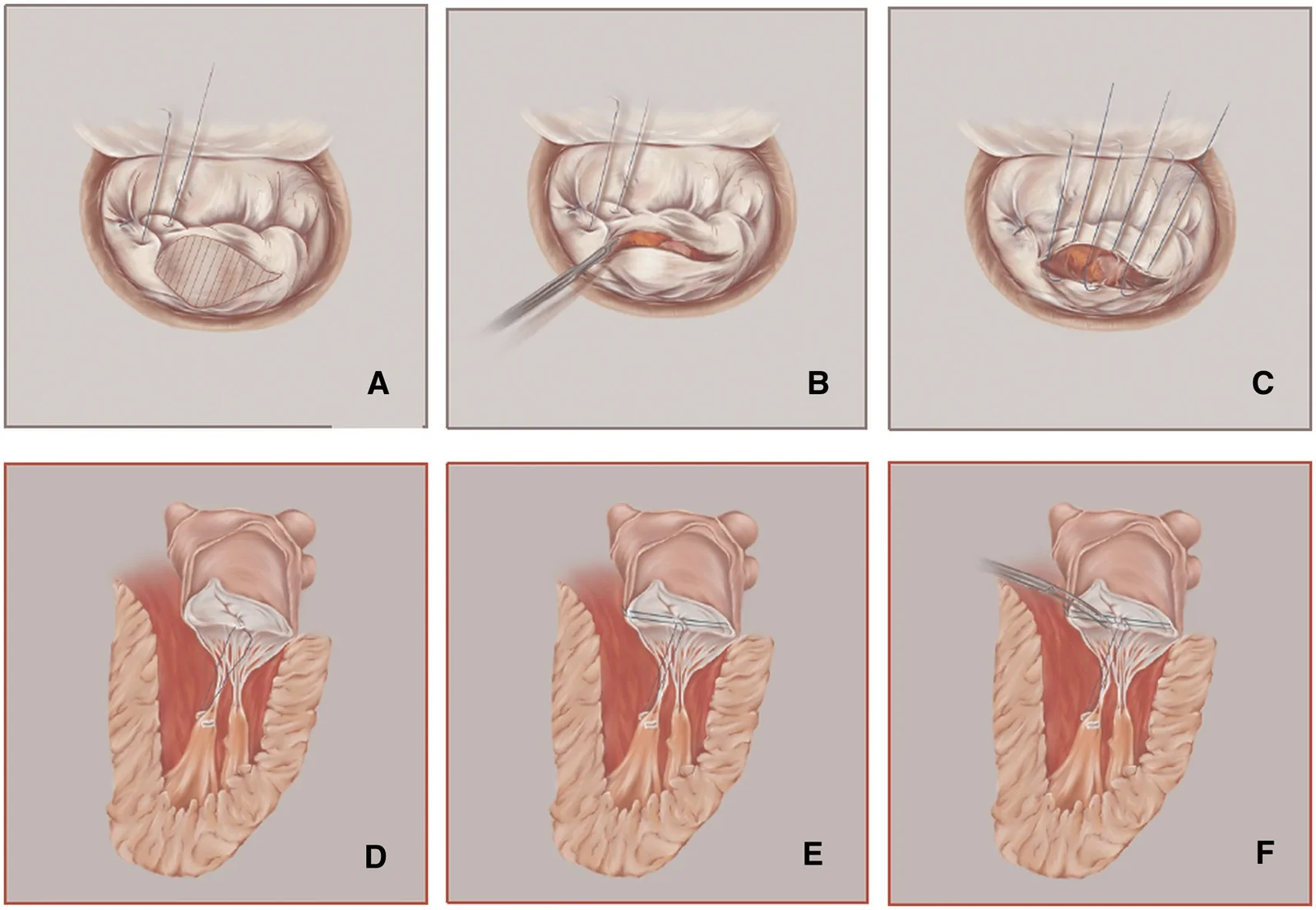
The RE-LOCK and TRACK Technique. (A-C) RE-LOCK, en-face view: target posterior segment; relocation of the redundant head to the new coaptation point; locking sutures on the correct plane. (D-F) TRACK, sectional left-ventricular view: neochord positioning; calibration of its length against the annular reference; final competent valve.

## Exposure and mechanism assessment

Through a right anterolateral mini-thoracotomy with peripheral femoral cannulation under transoesophageal guidance, the valve is exposed via a left atriotomy and inspected segment by segment. Distinguishing true anterior prolapse from anterior billowing or anterior override governs the entire operation. True prolapse shows the anterior free edge riding above the annular plane in systole, with an atrialized coaptation point and a posteriorly directed eccentric jet. In billowing, the body arches above the annulus while the free edge and coaptation point remain below it, with a central or absent jet; in override, a tall posterior scallop carries the coaptation line anteriorly without anterior elongation. This impression is confirmed on the arrested heart with a nerve hook, referenced to an adjacent non-prolapsing segment. The determination is provisional by design. Anterior neochordae are never committed on the preoperative study alone: the posterior platform is built first, and the anterior leaflet is then re-tested against it. Billowing and override resolve once posterior height is corrected and receive no neochordae; only a free edge still riding above the annular plane after RE-LOCK proceeds to TRACK.

## RE-LOCK—the posterior coaptation platform

The redundant or prolapsing posterior segment—usually P2, with variable extension to adjacent scallops—is identified and a stable posterior reference selected, typically the base of a non-prolapsing segment. Stay sutures on the body of the segment expose the coaptation surface and allow the target area to be defined without traction on the free margin. The redundant posterior head is then relocated towards the annular plane and anchored to this reference with fine interrupted polypropylene sutures, converting a mobile prolapsing edge into a controlled hinge and drawing the coaptation line back to a posteriorized position.^2^ Residual height is refined by folding, plication or a sliding plasty, preserving leaflet tissue rather than resecting it.^3^ Posterior neochordae are deliberately avoided in this substrate: the posterior problem is excess height rather than chordal length, and neochordae reposition the free edge while leaving the redundant body in place, so the coaptation line remains anterior and the risk of systolic anterior motion persists. We reserve them for the converse situations—a short posterior leaflet (P2 height below 10 mm), in which relocation would restrict, and isolated commissural prolapse. Adequacy is judged by saline testing, posterior symmetry and height—not by the volume of tissue removed.

## TRACK—anterior support, calibrated to the annulus

With posterior geometry fixed, anterior support is set by the TRACK concept, which references neochordal height to a temporary annular guide rather than to visual estimation or ventricular loading.^4^^,^^5^ CV-4 expanded polytetrafluoroethylene neochordae are anchored in the fibrous head of the appropriate papillary muscle, reproducing the native chordal vector, centred on A2 with extension to A1 or A3 as required; the free arms are passed through the anterior leaflet approximately 5 mm from the free edge with symmetric spacing, since bites placed too close to the margin risk tearing and asymmetric tension. A temporary annular guide, kept coplanar with the true annular plane, allows the anterior edge to be advanced to a reproducible reference height; the neochordal length is locked at that level and the guide removed. The aim is anterior competence without restriction and broad apposition against the RE-LOCK shelf.

## Ring annuloplasty and the rationale for the sequence

A complete rigid or semi-rigid annuloplasty ring is implanted as the final stabilizing step, sized to anterior leaflet dimensions and intercommissural distance; we do not downsize deliberately, since competence is obtained from leaflet geometry rather than from annular reduction. Concomitant tricuspid annuloplasty is added for at least moderate tricuspid regurgitation or significant annular dilatation. An alternative order—posterior repair, a true-sized ring, and only then a decision on anterior chordae—is entirely defensible, and in many bileaflet valves posterior repair with a ring is sufficient. We implant the ring last for 2 reasons. First, the ring should record a coaptation geometry that has already been established rather than create it; if it is placed before anterior calibration, any residual anterior deficit is corrected against a fixed annulus, and the temptation is to downsize. Second, subvalvular exposure through a mini-thoracotomy is appreciably better before the ring is seated. Final transoesophageal echocardiography confirms leaflet motion, a low transmitral gradient, broad coaptation and absence of systolic anterior motion, the decision resting on the global result rather than on coaptation length alone.

## Why it works

The strength of the protocol is its order. By fixing the sequence—posterior platform first, anterior calibration second—it converts individually demanding manoeuvres into a repeatable operation. Because both components are anatomy-based and reference-guided rather than estimation-based, they lend themselves to teaching and video-based review. In our experience, the protocol is reproducible, while the durability of its individual components has been reported separately.

## Data Availability

No new datasets were generated for this article; the clinical outcomes supporting the technique are reported separately.

## References

[1] Carpentier A. Cardiac valve surgery: the French correction. J Thorac Cardiovasc Surg. 1983;86:323-337. 6887954

[2] Speziale G , Moscarelli M, Di Bari N, et al A simplified technique for complex mitral valve regurgitation by a minimally invasive approach. Ann Thorac Surg. 2018;106:728-734. 29750930 10.1016/j.athoracsur.2018.04.010

[3] Perier P , Hohenberger W, Lakew F, et al Toward a new paradigm for the reconstruction of posterior leaflet prolapse: midterm results of the “respect rather than resect” approach. Ann Thorac Surg. 2008;86:718-725. 18721552 10.1016/j.athoracsur.2008.05.015

[4] Nasso G , Marchese A, Santarpino G, et al Annulus-guided neochord length setting for anterior mitral leaflet repair. Eur J Cardiothorac Surg. 2026;68:ezag025. 41546376 10.1093/ejcts/ezag025 PMC12957931

[5] Nasso G , Di Bari N, Bonifazi R, et al A new technique to adjust the length of artificial chordae during mitral anterior leaflet repair. J Card Surg. 2022;37:4517-4523. 36335612 10.1111/jocs.17108

